# Frontal Lobe and Subregional Volumetric Alterations Across Alzheimer’s Disease, Amnestic Mild Cognitive Impairment, and Vascular Dementia: An MRI Volumetry Study

**DOI:** 10.3390/brainsci16030317

**Published:** 2026-03-16

**Authors:** Stefan Stojanoski, Katarina Karher, Duško Kozić, Siniša S. Babović, Miloš Vuković, Katarina Koprivšek

**Affiliations:** Faculty of Medicine, University of Novi Sad, 21000 Novi Sad, Serbia; 015112@mf.uns.ac.rs (K.K.); dusko.kozic@mf.uns.ac.rs (D.K.); sinisa.babovic@mf.uns.ac.rs (S.S.B.); milos.vukovic@mf.uns.ac.rs (M.V.); katarina.koprivsek@mf.uns.ac.rs (K.K.)

**Keywords:** Alzheimer’s disease, amnestic mild cognitive impairment, vascular dementia, frontal lobe, brain volumetry, MRI, automated brain segmentation, volBrain

## Abstract

Background: Frontal lobe involvement represents an important but heterogeneously expressed feature across neurodegenerative and vascular cognitive disorders. While frontal atrophy has been described in Alzheimer’s disease (AD), detailed volumetric assessment of frontal subregions across Alzheimer’s disease, amnestic mild cognitive impairment (aMCI), and vascular dementia (VaD) remains insufficiently characterized. The aim of this study was to evaluate frontal lobe and frontal subregional volumetric alterations across these diagnostic groups using automated MRI-based volumetry. Methods: This cross-sectional study included 120 participants divided into four groups: AD, VaD, aMCI, and cognitively healthy controls (n = 30 per group). All participants underwent standardized neuropsychological assessment and 3T brain MRI. Automated volumetric analysis of the frontal lobe and its subregions was performed using the Vol2Brain pipeline. Group differences in total intracranial volume–adjusted frontal volumes were assessed using analysis of covariance, controlling for age and sex, followed by Bonferroni-corrected post hoc comparisons. False discovery rate (FDR) correction was applied across subregional comparisons. Results: A significant main effect of diagnostic group was observed for total frontal lobe volume, with lower adjusted volumes in patients with AD compared with aMCI and cognitively healthy controls. After correction for multiple comparisons, only total frontal lobe volume remained statistically significant. At the nominal level, group differences were observed in several frontal subregions, predominantly involving prefrontal and orbitofrontal areas. However, these findings did not survive FDR correction and should be interpreted as exploratory. No consistent frontal volumetric pattern was observed in VaD. Receiver operating characteristic analysis demonstrated moderate discriminatory ability of total frontal lobe volume for distinguishing AD from cognitively healthy controls. Conclusions: Automated MRI-based volumetry revealed global frontal lobe reduction in Alzheimer’s disease, whereas subregional findings were exploratory after correction for multiple testing. Frontal volumetric measures did not demonstrate a characteristic pattern in VaD. Global frontal volume may provide complementary structural information within clinically define cognitive disorders.

## 1. Introduction

Dementia represents a major global public health challenge and is among the leading causes of mortality worldwide. It is a progressive and irreversible neurodegenerative condition characterized by a decline in multiple cognitive domains, ultimately resulting in loss of functional independence. Approximately 57 million people are currently living with dementia globally, and this number is projected to exceed 150 million by 2050 [1]. The growing prevalence of dementia, together with its profound impact on patients, families, and healthcare systems, has intensified research efforts aimed at identifying risk factors, enabling early detection, and developing strategies to delay disease onset and progression [2]. The Lancet Commission on dementia prevention, intervention, and care has reported that up to 40% of dementia cases could potentially be prevented or delayed through modification of known risk factors [3]. Nevertheless, improving understanding of the disease etiology and refining diagnostic approaches remain central goals in dementia research. Within the diagnostic algorithm, advanced neuroimaging techniques, particularly structural magnetic resonance imaging (MRI), play a pivotal role in detecting brain changes at early stages, often before overt clinical manifestations become evident. Among the various dementia subtypes, Alzheimer’s disease (AD) and vascular dementia (VaD) represent the most prevalent and clinically significant entities.

Structural magnetic resonance imaging (MRI) is a fundamental component of dementia assessment, enabling in vivo evaluation of brain atrophy. Quantitative volumetric analysis provides objective and reproducible measurements of parenchymal loss and increases sensitivity to subtle morphometric changes [4,5]. The adoption of automated segmentation pipelines has facilitated large-scale volumetric studies by reducing operator dependency and the analysis time [6,7]. These advances have positioned automated MRI-based volumetry as an important tool for investigating disease-related patterns of neurodegeneration.

The frontal lobe plays a central role in executive control, attention, working memory, and behavioral regulation [8]. Impairment of these domains is common across cognitive disorders. Evidence indicates that frontal structures are affected in both AD and VaD, although the extent and distribution of involvement may differ between these entities [9,10]. Vascular pathology frequently disrupts frontal–subcortical networks, whereas AD is associated with more diffuse cortical degeneration that increasingly involves frontal regions as the disease progresses [11,12]. These observations highlight the frontal lobe as a clinically relevant target for quantitative neuroimaging investigations in dementia.

Although volumetric MRI studies have substantially advanced understanding of brain atrophy in dementia, most of the existing literature has primarily focused on medial temporal lobe structures, particularly the hippocampus, as key imaging biomarkers [13,14,15]. In contrast, frontal lobe involvement has received comparatively less detailed subregional investigation and is often treated as a composite region, rather than examined at the subregional level [16,17]. This approach may obscure disease-specific patterns within distinct frontal subregions, such as orbital, medial, and lateral frontal areas or motor-related cortices. Furthermore, only a limited number of studies have performed detailed subregional frontal volumetric analyses [18]. Direct volumetric comparisons across Alzheimer’s disease, mild cognitive impairment, and vascular dementia within a unified automated MRI-based framework and single cohort remain limited. In particular, few studies have systematically contrasted region-specific frontal vulnerability patterns across these clinically defined entities under identical methodological conditions. As a result, it remains unclear whether frontal involvement differs primarily in magnitude, in distribution, or in both across AD, aMCI and VaD.

Accordingly, the aim of the present study was to perform a comprehensive volumetric analysis of the frontal lobe and its subregions in patients with AD, amnestic mild cognitive impairment (aMCI), and VaD, compared with cognitively healthy controls. An automated MRI-based segmentation pipeline was used, and all regional volumes were normalized to total intracranial volume. We hypothesized that patients with AD would demonstrate the most pronounced global and subregional volume reductions, that aMCI would show milder and more selective alterations relative to AD, with partial preservations compared with controls, and that VaD would exhibit a different pattern of frontal involvement compared with AD, reflecting distinct underlying pathophysiological mechanisms rather than a simple gradient of atrophy.

## 2. Materials and Methods

This cross-sectional study was approved by the Ethics Committee of the Faculty of Medicine, University of Novi Sad, Serbia. Written informed consent was obtained from all participants prior to inclusion in the study.

### 2.1. Participants

A total of 120 participants were included and divided into four groups (n = 30 per group):

1. The Alzheimer’s disease (AD) group consisted of 30 participants (8 males, 22 females; mean age 73.3 ± 6.0 years) diagnosed with probable AD according to the National Institute on Aging–Alzheimer’s Association (NIA-AA) criteria [19].
2. The vascular dementia (VaD) group consisted of 30 participants (12 males, 18 females; mean age 67.9 ± 7.0 years) diagnosed with probable subcortical VaD according to the National Institute of Neurological Disorders and Stroke—Association Internationale pour la Recherche et l’Ensignement en Neurosciences (NINDS-AIREN) criteria, supported by clinical assessment and MRI findings [20].
3. The amnestic mild cognitive impairment (aMCI) group consisted of 30 participants (7 males, 23 females; mean age 69.3 ± 7.0 years) diagnosed with aMCI according to the NIA-AA criteria [19].
4. The control group consisted of 30 cognitively healthy individuals (9 males, 21 females; mean age 69.7 ± 4.8 years) with no history or clinical evidence of neurodegenerative, vascular, inflammatory, infectious, or metabolic brain disease, as well as no history of significant head trauma. The absence of pathology was additionally confirmed by neuropsychological assessment and brain MRI.

All clinical diagnoses were established by an experienced neurologist specializing in cognitive disorders, based on established diagnostic criteria, comprehensive neurological examinations, detailed neuropsychological assessments, and structural MRI findings to exclude alternative causes of cognitive impairment.

### 2.2. Neuropsychological Assessment

All participants underwent comprehensive neuropsychological assessment performed by an experienced clinical psychologist. Tests were administered and scored according to standardized procedures. Global cognitive functioning was evaluated using the Mini-Mental State Examination (MMSE) and the Addenbrooke’s Cognitive Examination Revised (ACE-R) [21,22]. Language abilities were assessed with the Boston Naming Test (BNT) and verbal fluency tasks [23]. Visuospatial abilities and visual organization were evaluated using the Hooper Visual Organization Test (HVOT) and the Rey–Osterrieth Complex Figure Test (ROCFT) [24,25]. Executive functions and attention were assessed with the Trail Making Test parts A and B (TMT-A, TMT-B), the Wisconsin Card Sorting Test (WCST), and the Executive Interview (EXIT) [26,27,28]. Verbal learning and memory were assessed using the Rey Auditory Verbal Learning Test (RAVLT) and the Wechsler Memory Scale–Revised (WMS-R) [29,30]. Depressive symptoms and behavioral changes were evaluated using the Beck Depression Inventory (BDI) and the Neuropsychiatric Inventory (NPI) [31,32]. Neuropsychological findings were used to support clinical diagnoses and group classification.

### 2.3. MRI Acquisition and Volumetric Analysis

All participants underwent brain MRI on a 3T scanner (Siemens Magnetom Trio, Erlangen, Germany) using a standard 64-channel head coil. The conventional protocol included T1-weighted, T2-weighted, FLAIR (fluid attenuated inversion recovery), and DWI (diffusion weighted imaging) sequences for diagnostic evaluation. For volumetric analysis, a high-resolution three-dimensional T1-weighted magnetization-prepared rapid gradient-echo (3D T1W MPRAGE) sequence with isotropic voxels and no interslice gap was acquired (TR/TE = 1530/2.97 ms, slice thickness 1 mm, field of view 25.8 cm, matrix 256 × 256, acquisition time 5:12 min). Only the 3D T1-weighted MPRAGE sequence was used for automated volumetric processing.

Volumetric analysis of brain structures was performed using the volBrain system, an automated online platform for segmentation and quantification of brain structures based on three-dimensional T1-weighted MRI scans (https://volbrain.net) [33]. For volumetric processing, three-dimensional T1-weighted MPRAGE images of all participants were converted from DICOM (Digital Imaging and Communications in Medicine) format to NIfTI (Neuroimaging Informatics Technology Initiative) format using the dcm2nii tool included in the MRIcron software package (version 1.0.20190902, accessed 2 September 2019, https://www.nitrc.org/projects/mricron/), as required by the volBrain platform.

**Figure 1:**
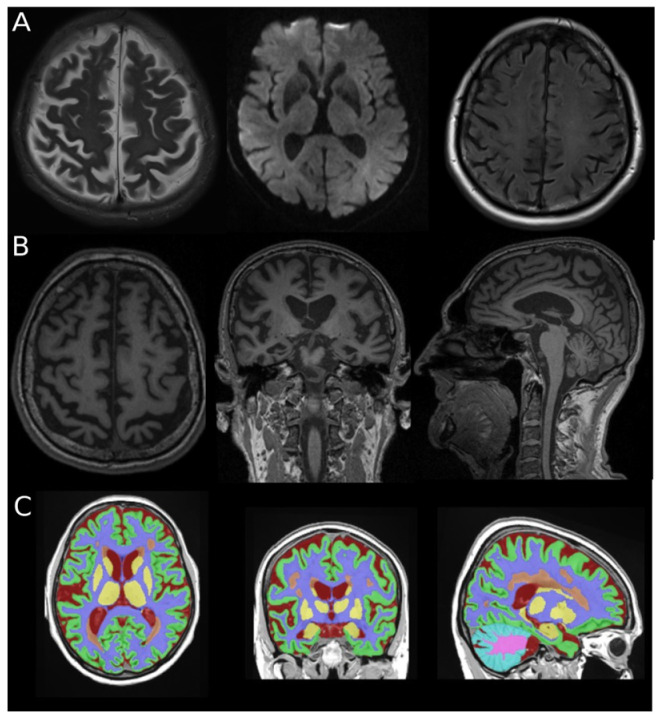
Example of MRI protocol and vol2Brain analysis. Images are provided for methodological illustration only and were not used for quantitative analyses. (**A**) T2-weighted, DWI and FLAIR. (**B**) 3D T1-weighted MPRAGE displayed in axial, coronal and sagittal planes. (**C**) Example of vol2Brain segmentation.

Image processing and segmentation were performed using the Vol2Brain pipeline and included the following steps: noise reduction (denoising), correction of intensity inhomogeneity (bias field correction), registration to the Montreal Neurological Institute (MNI) standard space, skull stripping, tissue classification into gray matter, white matter, and cerebrospinal fluid, intensity normalization, and anatomical segmentation [34,35,36,37,38]. Anatomical segmentation is based on a combination of multi-atlas labeling and patch-based methods, enabling parcellation of the brain into 135 anatomically defined regions [39,40]. For each segmented structure, relative volumes normalized to total intracranial volume (TIV) were calculated. The total intracranial volume was automatically estimated by the Vol2Brain pipeline during segmentation processing and used for normalization of regional volumes. The Vol2Brain pipeline performs automated tissue classification and atlas parcellation based on T1-weighted signal characteristics. White matter hyperintensities are not explicitly segmented as separate lesions within this framework but are incorporated into the automated tissue classification process. An illustrative example of the MRI protocol and automated segmentation is shown in Figure 1. Automated segmentation outputs generated by the volBrain platform were visually inspected using the system generated reports to confirm anatomical plausibility and the absence of gross tissue misclassification. No cases required exclusion due to segmentation failure.

Vol2Brain was selected due to its fully automated cloud-based processing, minimal operator intervention, and previously reported validation against established neuroimaging pipelines. The average processing time per case was approximately 15–20 min, depending on the server workload.

In the present study, volumetric measures were extracted for the total frontal lobe and the following frontal subregions: frontal pole, gyrus rectus, superior frontal gyrus (including medial segment), middle frontal gyrus, inferior frontal gyrus subregions (opercular, orbital, and triangular parts), anterior, posterior, lateral, and medial orbital gyri, precentral gyrus (including medial segment), supplementary motor cortex, and subcallosal area.

### 2.4. Statistical Analysis

Statistical analyses were performed using IBM SPSS Statistics software (version 27.0.1, IBM Corp., Armonk, NY, USA). The normality of distribution of the volumetric parameters was assessed using the Shapiro–Wilk test and visual inspection of histograms, box plots, and stem-and-leaf plots. The homogeneity of variances was evaluated using Levene’s test.

Group differences in demographic characteristics were analyzed using one-way analysis of variance (ANOVA) for continuous variables and the chi-square test for categorical variables. Comparisons of relative volumes of the examined brain structures between diagnostic groups were performed using analysis of covariance (ANCOVA), with age and sex included as covariates in all models. Relative volumetric measures normalized to total intracranial volume (TIV) were used for all analyses. The total intracranial volume was not entered as an additional covariate in the ANCOVA models, as all regional volumes were already normalized to the TIV.

When a significant main effect of a diagnostic group was observed, Bonferroni-corrected post hoc tests were applied to identify pairwise group differences. The effect size was expressed as the partial eta squared (η^2^). According to conventional benchmarks, partial eta squared values of approximately 0.01, 0.06 and 0.14 are considered small, medium and large effects respectively. The level of statistical significance was set at *p* < 0.05. The detailed results of covariate effects and post hoc comparisons are provided in the Supplementary Materials.

Receiver operating characteristic (ROC) analyses were performed to assess the discriminatory performance of the total frontal lobe volume. ROC curves were constructed for AD versus cognitively healthy controls and AD versus vascular dementia (VaD). Area under the curve (AUC) values with 95% confidence intervals were calculated using nonparametric methods.

To control for multiple comparisons across frontal subregional analyses, the Benjamini–Hochberg false discovery rate (FDR) procedure was applied (q = 0.05). The total frontal lobe volume was defined as the primary outcome measure, while subregional analyses were treated as exploratory.

## 3. Results

### 3.1. Participant Characteristics

A total of 120 participants were included (AD, VaD, aMCI, and cognitively healthy controls; n = 30 per group). A significant difference in age was observed among groups (one-way ANOVA, F(3,114) = 3.536, *p* = 0.017), whereas the sex distribution did not differ significantly (χ^2^(3) = 2.22, *p* = 0.528). Accordingly, all volumetric comparisons were performed using ANCOVA models including age and sex as covariates in all analyses. The demographic data are summarized in Table 1.

### 3.2. Frontal Lobe Volumetry

**Figure 2:**
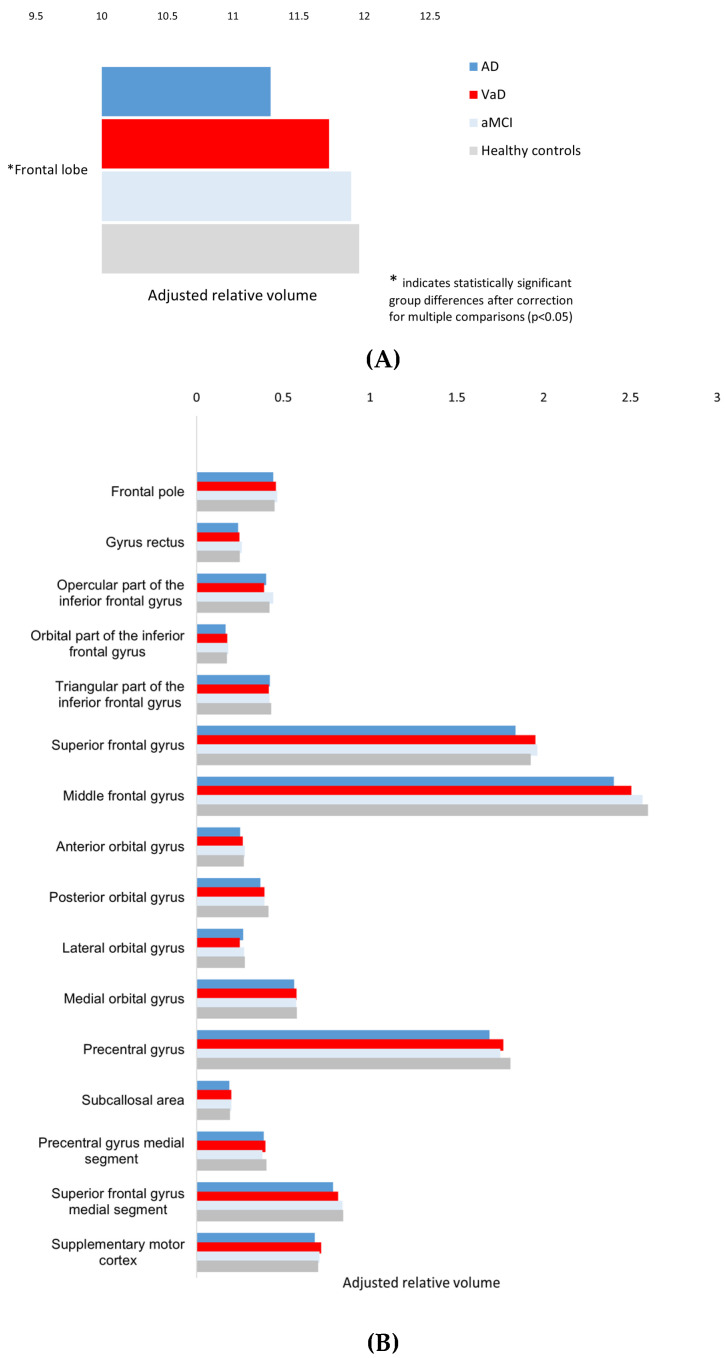
Estimated marginal means of relative volumes for the frontal lobe and its subregions across diagnostic groups, adjusted for age and sex. (**A**) Adjusted mean total frontal volumes. Figure (**B**) Adjusted mean volumes of frontal subregions.

Overall, nominal main effects of diagnostic group were detected for the total frontal lobe volume and several frontal subregions. Group-level patterns across frontal lobe volumes are illustrated in Figure 2A, while Figure 2B presents the distribution of the adjusted volumes across frontal subregions.

The main volumetric results are presented in Table 2, with detailed Bonferroni-adjusted pairwise post hoc comparisons provided in Supplementary Table S1, and the effects of age and sex are reported in Supplementary Table S2. Ninety-five percent confidence intervals for estimated marginal means and group differences are provided in Supplementary Table S3.

After controlling for multiple comparisons using the Benjamini–Hochberg procedure (*q* = 0.05), only the total frontal lobe volume remained statistically significant. Accordingly, subregional findings should be interpreted as exploratory.

A significant main effect of diagnostic group was observed for the total frontal lobe volume, with lower volumes in patients with Alzheimer’s disease compared with individuals with aMCI and cognitively healthy controls (Table 2; Supplementary Table S1). No significant differences were detected between the VaD group and AD and cognitively healthy controls.

At the nominal level (uncorrected ANCOVA main effects), group differences were observed in several frontal subregions. Within the orbitofrontal cortex, nominal effects were detected for the anterior and posterior orbital gyri. Volumes of the anterior orbital gyrus were higher in the aMCI group compared with AD, while posterior orbital gyrus volumes were lower in AD relative to cognitively healthy controls. However, these findings did not remain significant after FDR correction.

Similarly, nominal group effects based on uncorrected comparisons were observed for the opercular part of the inferior frontal gyrus, as well as for the superior frontal gyrus, middle frontal gyrus, and precentral gyrus. In these regions, AD was generally associated with lower adjusted volumes compared to cognitively healthy controls and in some instances to aMCI. These subregional differences did not survive FDR correction and should therefore be interpreted cautiously.

No group differences were observed for the frontal pole, gyrus rectus, triangular or orbital parts of the inferior frontal gyrus, lateral of medial orbital gyri, medial segment of the superior or precentral gyri, supplementary motor cortex, or subcallosal area.

### 3.3. Discriminator Performance of Total Frontal Lobe Volume

Receiver operating characteristic analysis demonstrated that the total frontal lobe volume significantly discriminated AD from cognitively healthy controls (AUC = 0.773, 95% CI 0.653–0.892, *p* < 0.001).

In the comparison between AD and VaD, the total frontal lobe volume showed modest but statistically significant discriminatory ability (AUC = 0.677, 95% CI 0.539–0.814, *p* = 0.019).

Detailed ROC metrics are presented in Table 3.

## 4. Discussion

The present study examined subregional frontal lobe volumetric alterations across AD, aMCI, VaD, and cognitively healthy controls using automated MRI-based segmentation. At the global level, AD was associated with a significantly reduced frontal lobe volume compared with aMCI and controls, whereas VaD demonstrated relative preservation of the overall frontal volume. At the subregional level, nominal volumetric reductions in AD were observed in several prefrontal and orbitofrontal regions. However, these findings did not remain significant after correction for multiple comparisons.

In contrast, VaD did not demonstrate a consistent or regionally specific pattern of frontal volumetric involvement. Collectively, these findings suggest a pattern of regional involvement that warrants replication in larger cohorts. Formal discriminatory analysis demonstrated that the global frontal lobe volume represents the most robust and statistically supported marker in the present cohort.

Consistent with these findings, the frontal lobe volume differed significantly across diagnostic groups, with AD patients showing the lowest volumes. In contrast, aMCI demonstrated relative preservation of the overall frontal lobe volume, with values comparable to cognitively healthy controls. VaD did not show statistically significant differences in the frontal lobe volume compared with AD, aMCI, or cognitively healthy controls. This pattern is consistent with the predominantly cortical neurodegenerative nature of AD, whereas frontal involvement in VaD is thought to be mediated primarily through disruption of frontal–subcortical circuits rather than primary cortical degeneration [41,42]. Previous volumetric MRI studies have similarly reported frontal lobe volume reductions in AD, while frontal changes in aMCI are typically less pronounced and more variable across cohorts [43,44,45]. Together, these findings indicate that the global frontal lobe volumetry reflects the overall disease burden, which demonstrated statistically significant discriminatory ability in the present cohort. However, the effect sizes indicate that it should not be interpreted as a standalone diagnostic marker. Receiver operating characteristic analysis further demonstrated that the total frontal lobe volume significantly discriminated AD from cognitively healthy controls (AUC = 0.773) and showed modest but statistically significant discrimination between AD and VaD (AUC = 0.677). These findings indicate that global frontal atrophy carries measurable diagnostic information, although its discriminatory performance remains moderate.

Beyond global frontal lobe volume loss, nominal differences across several frontal subregions were observed in AD, suggesting a possible regionally patterned distribution. At the nominal level, lower adjusted volumes were observed in several prefrontal gyri, including the middle and superior frontal and the opercular part of the inferior frontal gyrus, as well in selected orbitofrontal regions, namely the anterior and posterior orbital gyri. Previous voxel-based and volumetric MRI studies have similarly reported atrophy of the middle and superior frontal gyri and inferior frontal regions in AD, supporting the preferential vulnerability of associative prefrontal cortices [46,47,48]. In addition, frontal volume reductions have been linked to neuropsychiatric and behavioral symptoms in AD, underscoring the clinical relevance of prefrontal degeneration [49]. Nominal differences involving anterior and posterior orbital gyri are compatible with previously reported orbitofrontal involvement, which is critically involved in behavioral regulation, decision-making, and socio-emotional processing [48,49]. Orbitofrontal atrophy has likewise been associated with neuropsychiatric manifestations and disease severity in AD [49]. Nominal differences in the precentral gyrus may reflect the extension of neurodegenerative changes toward motor and premotor networks or broader disruption of frontal network integrity in AD [50]. Collectively, these nominal findings are compatible with previously described patterns of prefrontal and orbitofrontal involvement, although they require confirmation in larger samples. Given the multiple-comparison burden, these findings should be interpreted as exploratory.

Compared with AD, aMCI was characterized by the relative preservation of frontal subregions, with only subtle and heterogeneous volumetric alterations. This cross-sectional pattern is compatible with established clinical staging frameworks in which aMCI is described as a transitional diagnostic category. However, the present design does not permit inference regarding longitudinal progression. In the present study, aMCI did not exhibit widespread frontal subregional atrophy and demonstrated only nominal differences relative to AD. At the nominal level, the differences between AD and aMCI were confined to the superior frontal gyrus, whereas differences in the middle frontal gyrus were primarily observed between AD and cognitively healthy controls. Notably, frontal volumes in aMCI were largely comparable to those of cognitively healthy controls, suggesting relative preservation of frontal structural integrity in this group. Previous MRI studies indicate that early structural changes in aMCI predominantly affect medial temporal lobe regions, whereas frontal involvement is less consistent and typically milder [51,52,53]. In particular, volumetric alterations of the entorhinal cortex have been reported in aMCI, underscoring the prominent involvement of medial temporal structures in the Alzheimer’s disease spectrum [54]. Nevertheless, voxel-based and surface-based morphometry studies have demonstrated that a subset of aMCI individuals may exhibit limited frontal cortical involvement, including the middle frontal gyrus and lateral orbital cortex [54,55,56]. This finding further supports the heterogeneity of the aMCI phenotype. Collectively, these nominal findings suggest that aMCI does not display a stable frontal subregional atrophy pattern. Subtle frontal alterations, when present, may reflect region-specific vulnerability patterns, as described in previous research.

In our cohort, the VaD group did not show significant differences in total frontal lobe volume compared with AD, aMCI, or cognitively healthy controls. Across frontal subregions, no consistent VaD-related volumetric reductions were detected relative to the other diagnostic groups. At the nominal level, a post hoc difference involving VaD was observed in the opercular part of the inferior frontal gyrus, where the aMCI group showed a higher relative volume than the VaD group, without evidence of a broader regional pattern. These findings indicate that frontal involvement in VaD is not characterized by a consistent pattern of cortical volumetric loss. Given that our VaD cohort consisted of patients with probable subcortical VaD according to NINDS-AIREN criteria, these findings may be compatible with current conceptual models of subcortical VaD, in which cognitive impairment is frequently attributed to the disruption of fronto-subcortical and cortico-thalamic networks rather than to primary cortical neurodegeneration [57,58]. However, as vascular lesion burden (e.g., white matter hyperintensities or lacunar infarcts) and other markers of small vessel disease were not quantitatively assessed in the present study, mechanistic interpretation should be considered speculative. Cortical thickness studies in subcortical vascular cognitive impairment and subcortical VaD have reported frontal cortical involvement, particularly within prefrontal and cingulate regions, as well as associations between frontal cortical integrity and executive or working memory performance [59,60]. However, our volumetric results suggest that such changes may not necessarily manifest as robust reductions in frontal subregional volumes.

Overall, our findings indicate that the diagnostic groups differ in the magnitude of frontal lobe involvement, while nominal differences suggest potential variation in subregional patterns. Alzheimer’s disease was characterized by the most consistent frontal volumetric reductions, whereas aMCI showed milder and more selective changes, and VaD did not demonstrate a robust frontal volumetric signature. Taken together, these results support the relevance of a global frontal volumetric reduction in AD, while nominal subregional patterns may offer complementary descriptive information that warrants replication in larger cohorts. These interpretations remain grounded in clinically defined diagnostic categories rather than biomarker-confirmed disease entities. In addition, the present study allows the direct comparison of AD, aMCI and VaD within a single cohort, using a standardized automated segmentation framework and uniform statistical modeling. This design supports the consistent evaluation of frontal involvement across clinically defined diagnostic groups under comparable methodological conditions.

### 4.1. Clinical Implications

The present findings suggest that, although only the global frontal lobe volume remains statistically robust after correction, subregional frontal lobe volumetry may provide complementary descriptive information regarding structural profiles across clinically defined etiologies of cognitive impairment, However, direct structure–function relationships with neuropsychological measures were not assessed in the present study. By applying the same automated volumetric pipeline across all diagnostic groups, the study reflects a framework that may be feasible in routine clinical research settings and supports reproducible structural assessment. Detailed frontal volumetric evaluation may complement clinical assessment when interpreted within a broader multimodal complex. Frontal volumetric measures should not be considered standalone diagnostic markers. Instead, they should be integrated with clinical presentation, cognitive testing, and conventional neuroimaging findings to support comprehensive patient evaluation.

### 4.2. Methodological Strengths

This study benefits from a standardized and fully automated MRI segmentation approach, which reduces operator dependence and enhances reproducibility. The use of a subregional frontal lobe analysis allows more detailed characterization of structural changes than global volumetric measures alone. In addition, normalization of regional volumes to intracranial volume improves the validity of inter-individual comparisons. Finally, the inclusion of well-defined diagnostic groups and the use of appropriate covariates strengthen the robustness of the presented findings. The use of a unified automated pipeline across all diagnostic groups further enhances methodological consistency and enables direct group comparison under identical analytical conditions.

### 4.3. Limitations

Several limitations of this study should be acknowledged. The relatively modest sample size and single-center design may limit the generalizability of the findings. In addition, the age differed significantly between groups at the univariate level. Although age was included as a covariate in all ANCOVA models, residual confounding related to age-related frontal volume decline cannot be entirely excluded. No a priori power calculation was performed. Although the group sizes were comparable to those reported in similar neuroimaging studies, the sample size may limit the statistical power to detect small to moderate subregional effects. Consequently, the possibility of both type I and type II errors cannot be fully excluded, particularly for exploratory subregional analyses. In addition, the cross-sectional nature of the study precludes the assessment of longitudinal changes in and trajectories of frontal volumetric alterations. The absence of molecular biomarkers (e.g., amyloid, tau, or CSF-based measures) limits biological validation of the clinical diagnoses. Although established diagnostic criteria were applied, the lack of biomarker confirmation introduces the possibility of misclassification, particularly within the aMCI group. Consequently, etiological inferences regarding disease-specific frontal patterns should be interpreted with caution. In addition, the vascular lesion burden (including white matter hyperintensities or lacunar infarcts) was not quantitatively evaluated, precluding direct assessment of small vessel disease severity and structural network disruption in the VaD group. Given the number of subregional comparisons performed, and despite application of FDR correction, the subregional findings should be interpreted as exploratory, particularly as only the global frontal measure remained significant after correction for multiple testing. Direct analyses linking frontal subregional volumes to neuropsychological performance were not performed. Such analyses could provide additional insight into the clinical and behavioral relevance of the observed structural patterns. Therefore, the clinical implication of subregional volumetric findings should be interpreted with appropriate caution. Finally, although a validated and automated segmentation pipeline was used, reliance on a single software tool may introduce method-specific effects, and future studies could benefit from cross-validation across different segmentation approaches.

### 4.4. Future Directions

Future studies should aim to validate these findings, particularly subregional patterns, in larger multicenter cohorts and to explore longitudinal changes in frontal subregional volumes across disease stages. The integration of multimodal imaging approaches, including diffusion- and functional-based techniques, may provide additional insight into network-level mechanisms underlying frontal dysfunction. Furthermore, combining subregional volumetric measures with neuropsychological and molecular biomarker data may help clarify the relationships between structural alterations and clinical phenotypes.

## 5. Conclusions

In summary, this study demonstrates that global frontal lobe involvement differs across clinically defined etiologies of cognitive impairment, with pronounced global frontal volumetric reductions in Alzheimer’s disease, predominantly nominal subregional changes in aMCI and the absence of consistent frontal volumetric loss in VaD. This pattern may partly reflect the heterogeneity of vascular lesion burden, distribution, and disease stage among individuals with VaD. These findings highlight the potential descriptive value of subregional frontal volumetric analysis in characterizing structural pattern across diagnostic groups, while underscoring the need for multimodal approaches to clarify the underlying neurobiological mechanisms. Overall, a detailed assessment of frontal subregions may contribute to a more integrated understanding of structural differences observed across major causes of cognitive impairment.

## Figures and Tables

**Table 1:** Demographic characteristics of study groups.

Group	AD	VaD	aMCI	Control	*p*-Value
**Number of subjects**	30	30	30	30	
**Age (mean ± SD)**	73.31 ± 6.03	68.13 ± 6.96	69.30 ± 6.97	69.73 ± 4.83	0.017
**Sex (M/F)**	8/22	12/18	7/23	9/21	0.528

**Table 2:** Volumes of total frontal lobe and frontal subregions across diagnostic groups.

STRUCTURE	Group	x	SD	Estimated x	SE	95% CI		F(3,114)	*p*	*η* ^2^
						Lower	Upper			
**Frontal lobe**	AD	11.22	0.92	11.287	0.132	11.026	11.548	5.387	0.002	0.124
	VaD	11.76	0.61	11.730	0.130	11.472	11.988			
	aMCI	11.92	0.73	11.900	0.128	11.646	12.154			
	Control	11.97	0.50	11.962	0.128	11.709	12.215			
**Frontal pole**	AD	0.436	0.056	0.441	0.010	0.422	0.461	1.029	0.382	0.026
	VaD	0.461	0.055	0.457	0.010	0.438	0.477			
	aMCI	0.467	0.056	0.465	0.010	0.446	0.485			
	Control	0.451	0.049	0.450	0.010	0.431	0.470			
**Gyrus rectus**	AD	0.237	0.026	0.240	0.005	0.230	0.250	2.418	0.070	0.060
	VaD	0.249	0.028	0.248	0.005	0.237	0.258			
	aMCI	0.260	0.030	0.260	0.005	0.249	0.270			
	Control	0.250	0.028	0.250	0.005	0.240	0.260			
**Opercular part of the inferior frontal gyrus**	AD	0.397	0.078	0.401	0.012	0.377	0.425	3.668	0.014	0.088
	VaD	0.392	0.051	0.389	0.012	0.365	0.413			
	aMCI	0.441	0.065	0.441	0.012	0.418	0.465			
	Control	0.420	0.063	0.420	0.012	0.396	0.443			
**Orbital part of the inferior frontal gyrus**	AD	0.167	0.038	0.167	0.008	0.151	0.183	0.636	0.593	0.016
	VaD	0.177	0.045	0.177	0.008	0.161	0.193			
	aMCI	0.183	0.035	0.182	0.008	0.167	0.198			
	Control	0.176	0.049	0.176	0.008	0.161	0.192			
**Triangular part of the inferior frontal gyrus**	AD	0.418	0.063	0.422	0.009	0.403	0.440	0.454	0.715	0.012
	VaD	0.418	0.042	0.416	0.009	0.397	0.434			
	aMCI	0.422	0.052	0.420	0.009	0.402	0.438			
	Control	0.432	0.044	0.431	0.009	0.412	0.449			
**Middle frontal gyrus**	AD	2.379	0.257	2.404	0.042	2.424	2.588	4.361	0.006	0.103
	VaD	2.520	0.230	2.506	0.042	2.321	2.487			
	aMCI	2.578	0.236	2.569	0.041	2.488	2.650			
	Control	2.603	0.182	2.601	0.041	2.520	2.682			
**Superior frontal gyrus**	AD	1.837	0.216	1.838	0.033	1.772	1.904	2.960	0.035	0.072
	VaD	1.951	0.135	1.953	0.033	1.888	2.018			
	aMCI	1.968	0.173	1.965	0.032	1.901	2.029			
	Control	1.925	0.166	1.925	0.032	1.862	1.989			
**Anterior orbital gyrus**	AD	0.250	0.038	0.251	0.006	0.253	0.279	3.409	0.020	0.082
	VaD	0.267	0.029	0.266	0.006	0.238	0.264			
	aMCI	0.279	0.039	0.279	0.006	0.266	0.291			
	Control	0.273	0.031	0.273	0.006	0.261	0.286			
**Posterior orbital gyrus**	AD	0.367	0.052	0.368	0.009	0.350	0.387	3.931	0.010	0.094
	VaD	0.392	0.048	0.391	0.009	0.372	0.409			
	aMCI	0.391	0.040	0.391	0.009	0.373	0.410			
	Control	0.414	0.057	0.414	0.009	0.395	0.432			
**Lateral orbital gyrus**	AD	0.264	0.057	0.268	0.009	0.250	0.287	1.954	0.125	0.049
	VaD	0.252	0.054	0.250	0.009	0.232	0.268			
	aMCI	0.276	0.037	0.275	0.009	0.257	0.293			
	Control	0.278	0.046	0.278	0.009	0.260	0.296			
**Medial orbital gyrus**	AD	0.560	0.057	0.562	0.009	0.544	0.581	0.618	0.605	0.016
	VaD	0.577	0.036	0.576	0.009	0.558	0.594			
	aMCI	0.576	0.049	0.575	0.009	0.558	0.593			
	Control	0.573	0.049	0.579	0.009	0.561	0.597			
**Precentral gyrus**	AD	1.685	0.180	1.688	0.028	1.631	1.745	3.061	0.031	0.075
	VaD	1.766	0.149	1.767	0.029	1.711	1.823			
	aMCI	1.753	0.138	1.750	0.028	1.695	1.805			
	Control	1.808	0.135	1.808	0.028	1.753	1.753			
**Subcallosal area**	AD	0.189	0.029	0.188	0.005	0.178	0.198	1.447	0.233	0.037
	VaD	0.201	0.019	0.201	0.005	0.191	0.211			
	aMCI	0.199	0.031	0.200	0.005	0.191	0.210			
	Control	0.193	0.027	0.193	0.005	0.184	0.203			
**Precentral gyrus medial segment**	AD	0.384	0.043	0.387	0.008	0.371	0.403	1.707	0.170	0.043
	VaD	0.399	0.050	0.397	0.008	0.381	0.413			
	aMCI	0.379	0.040	0.379	0.008	0.363	0.395			
	Control	0.402	0.040	0.402	0.008	0.386	0.418			
**Superior frontal** **gyrus medial segment**	AD	0.782	0.099	0.787	0.017	0.753	0.820	2.447	0.067	0.060
	VaD	0.819	0.099	0.816	0.017	0.783	0.850			
	aMCI	0.842	0.083	0.840	0.017	0.807	0.873			
	Control	0.844	0.077	0.844	0.016	0.811	0.876			
**Supplementary motor cortex**	AD	0.678	0.064	0.682	0.013	0.656	0.708	1.382	0.252	0.035
	VaD	0.723	0.073	0.719	0.013	0.693	0.745			
	aMCI	0.710	0.074	0.708	0.013	0.682	0.733			
	Control	0.702	0.071	0.701	0.013	0.676	0.726			

**Table 3:** ROC analysis of total frontal lobe volume.

Comparison	AUC	95% CI	*p*-Value
AD vs. control	0.773	0.653–0.892	<0.001
AD vs. VaD	0.677	0.539–0.814	0.019

## Data Availability

The human data supporting the findings of this study are not openly available due to privacy and sensitivity concerns. They are available from the corresponding author upon request.

## Supplementary Materials

The following supporting information can be downloaded at https://www.mdpi.com/article/10.3390/brainsci16030317/s1, Table S1: Post hoc Bonferroni analysis; Table S2: Effects of age and sex covariates; Table S3: Bonferroni-adjusted pairwise comparison with 95% CI for regions showing significant main effects in ANCOVA.

## Abbreviations

The following abbreviations are used in this manuscript:

3D	three-dimensional
ACE-R	Addenbrooke’s Cognitive Examination Revised
AD	Alzheimer’s disease
aMCI	amnestic mild cognitive impairment
ANCOVA	analysis of covariance
ANOVA	analysis of variance
BDI	Beck Depression Inventory
BNT	Boston Naming Test
DICOM	Digital Imaging and Communications in Medicine
DWI	diffusion-weighted imaging
EXIT	Executive Interview
FLAIR	fluid-attenuated inversion recovery
HVOT	Hooper Visual Organization Test
MNI	Montreal Neurological Institute
MPRAGE	magnetization-prepared rapid gradient-echo
MRI	magnetic resonance imaging
NIfTI	Neuroimaging Informatics Technology Initiative
NIA-AA	National Institute on Aging–Alzheimer’s Association
NINDS-AIREN	National Institute of Neurological Disorders and Stroke–Association Internationale pour la Recherche et l’Enseignement en Neurosciences
NPI	Neuropsychiatric Inventory
RAVLT	Rey Auditory Verbal Learning Test
ROCFT	Rey–Osterrieth Complex Figure Test
SD	standard deviation
TE	echo time
TIV	total intracranial volume
TMT-A/TMT-B	Trail Making Test parts A and B
TR	repetition time
VaD	vascular dementia
WCST	Wisconsin Card Sorting Test
WMS-R	Wechsler Memory Scale–Revised
