# Supplementary material for: Frontal Lobe and Subregional Volumetric Alterations Across Alzheimer’s Disease, Amnestic Mild Cognitive Impairment, and Vascular Dementia: An MRI Volumetry Study
Source: Brain Sci. 2026 Mar 16;16(3):317. doi: 10.3390/brainsci16030317 (PMC13024419; doi:10.3390/brainsci16030317)
Supplement: Supplementary file 1 [file brainsci-16-00317-s001.zip › brainsci-4175354-supplementary.pdf]

Supplementary Table S1. Post hoc Bonferroni analysis.

	**AD vs VaD**	**AD vs aMCI**	**AD vs control**	**VaD vs aMCI**	**VaD vs control**	**aMCI vs control**
**Frontal lobe**	0.125	**0.007**	**0.002**	1.000	1.000	1.000
**Frontal pole**	1.000	0.562	1.000	1.000	1.000	1.000
**Gyrus rectus**	1.000	0.054	1.000	0.628	1.000	1.000
**Opercular part of inferior frontal gyrus**	1.000	0.122	1.000	**0.016**	0.425	1.000
**Orbital part of inferior frontal gyrus**	1.000	1.000	1.000	1.000	1.000	1.000
**Triangular part of inferior frontal gyrus**	1.000	1.000	1.000	1.000	1.000	1.000
**Middle frontal gyrus**	0.556	**0.036**	**0.006**	1.000	0.628	1.000
**Superior frontal gyrus**	0.101	**0.043**	0.371	1.000	1.000	1.000
**Anterior orbital gyrus**	0.728	**0.019**	0.106	0.914	1.000	1.000
**Posterior orbital gyrus**	0.611	0.500	**0.005**	1.000	0.490	0.546
**Lateral orbital gyrus**	0.976	1.000	1.000	0.301	0.176	1.000
**Medial orbital gyrus**	1.000	1.000	1.000	1.000	1.000	1.000
**Precentral gyrus**	0.339	0.758	**0.020**	1.000	1.000	0.857
**Subcallosal area**	0.472	0.539	1.000	1.000	1.000	1.000
**Precentral gyrus- medial segment**	1.000	1.000	1.000	0.629	1.000	0.243
**Superior frontal gyrus-medial segment**	1.000	0.163	0.108	1.000	1.000	1.000
**Supplementary motor cortex**	0.298	0.976	1.000	1.000	1.000	1.000

Supplementary Table S2. Effects of age and sex covariates.

	**Age (*p*)**	**Sex (*p*)**
**Frontal lobe**	**0.034**	0.513
**Frontal pole**	**0.035**	0.816
**Gyrus rectus**	**0.026**	0.587
**Opercular of inferior frontal gyrus**	0.239	0.466
**Orbital of inferior frontal gyrus**	0.993	0.696
**Triangular of inferior frontal gyrus**	0.062	0.352
**Middle frontal gyrus**	**0.013**	0.477

**Superior frontal gyrus**	0.786	0.336
**Anterior orbital gyrus**	0.468	0.455
**Posterior orbital gyrus**	0.768	0.509
**Lateral orbital gyrus**	0.103	0.781
**Medial orbital gyrus**	0.223	0.816
**Precentral gyrus**	0.547	0.206
**Subcallosal area**	0.246	0.112
**Precentral gyrus-medial segment**	0.175	0.908
**Superior frontal gyrus-medial segment**	0.240	0.689
**Supplementary motor cortex**	0.147	0.880

Supplementary Table S3. Bonferroni-adjusted pairwise comparison with 95% CI for regions showing significant main effects in ANCOVA.

Structure	Comparison	Mean difference	95% CI lower	95% CI upper	*p* (Bonf. corrected)
**Total frontal lobe**	AD vs VaD	-0.443	-0.950	0.065	0.125
	AD vs aMCI	-0.613	-0.950	0.065	**0.007**
	AD vs control	-0.674	-1.169	-0.180	**0.002**
	VaD vs aMCI	-0.170	-0.660	0.320	1.000
	VaD vs control	-0.232	-0.720	0.257	1.000
	aMCI vs control	-0.062	-0.547	0.424	1.000
**Opercular inferior frontal gyrus**	AD vs VaD	0.012	-0.035	0.059	1.000
	AD vs aMCI	-0.040	-0.086	0.006	0.122
	AD vs control	-0.019	-0.086	0.006	1.000
	VaD vs aMCI	-0.052	-0.098	-0.007	**0.016**
	VaD vs control	-0.031	-0.076	0.015	0.425
	aMCI vs control	0.021	-0.024	0.066	1.000

**Middle frontal gyrus**	AD vs VaD	-0.102	-0.264	0.060	0.556
	AD vs aMCI	-0.165	-0.323	-0.007	**0.036**
	AD vs control	-0.197*	-0.355	-0.040	**0.006**
	VaD vs aMCI	-0.063	-0.219	0.094	1.000
	VaD vs control	-0.095	-0.251	0.061	0.628
	aMCI vs control	-0.032	-0.187	0.123	1.000
**Superior frontal gyrus**	AD vs VaD	-.115	-0.243	0.012	0.101
	AD vs aMCI	-.127	-0.252	-0.003	**0.043**
	AD vs control	-.087	-0.211	0.037	0.371
	VaD vs aMCI	-.012	-0.135	0.111	1.000
	VaD vs control	.028	-0.095	0.151	1.000
	aMCI vs control	.040	-0.082	0.162	1.000
**Anterior orbital gyrus**	AD vs VaD	-0.015	-0.040	0.010	0.728
	AD vs aMCI	-0.028	-0.052	-0.003	**0.019**
	AD vs control	-0.022	-0.046	0.003	0.106
	VaD vs aMCI	-0.013	-0.037	0.011	0.914
	VaD vs control	-0.007	-0.031	0.017	1.000
	aMCI vs control	0.006	-0.018	0.030	1.000
**Posterior orbital gyrus**	AD vs VaD	-0.022	-0.059	0.014	0.611
	AD vs aMCI	-0.023	-0.059	0.012	0.500
	AD vs control	-0.045	-0.081	-0.010	**0.005**
	VaD vs aMCI	-0.001	-0.036	0.034	1.000
	VaD vs control	-0.023	-0.058	0.012	0.490
	aMCI vs control	-0.022	-0.057	0.013	0.546

**Precentral gyrus**	AD vs VaD	-0.079	-0.189	0.031	0.339
	AD vs aMCI	-0.062	-0.169	0.046	0.758
	AD vs control	-0.120	-0.227	-0.012	**0.020**
	VaD vs aMCI	0.017	-0.089	0.124	1.000
	VaD vs control	-0.041	-0.147	0.065	1.000
	aMCI vs control	-0.058	-0.163	0.047	0.857
